# Modeling Overall Survival in Patients With Pancreatic Cancer From a Pooled Analysis of Phase II Trials

**DOI:** 10.1002/cam4.70289

**Published:** 2024-10-10

**Authors:** Eva Rahman Kabir, Faruque Azam, Tanisha Tabassum Sayka Khan, Hasina Yasmin, Namara Mariam Chowdhury, Syeda Maliha Ahmed, Baejid Hossain Sagar, Nasrin Ahmed Tahrim

**Affiliations:** ^1^ School of Pharmacy BRAC University Dhaka Bangladesh

**Keywords:** clinical trials, overall survival, pancreatic adenocarcinoma, predictive survival modeling, progression‐free survival, response rate

## Abstract

**Background:**

We evaluated the validity of surrogacy of progression‐free survival (PFS) or time‐to‐progression (TTP) and overall response rate (ORR) in phase II trials of pancreatic ductal adenocarcinoma (PDAC). In addition, we explored the impact of predictive variables on overall survival (OS) and developed an optimal OS model.

**Methods:**

We analyzed 1867 clinical endpoint from 619 phase II PDAC trials with a systematic search from PubMed. Endpoint correlations were determined by Spearman's rank correlation. The assessed predictive factors included PFS/TTP, treatment size, therapy type, stage, and previous treatment. The relationship between predictors and OS was explored by a gamma generalized linear model (GLM) with a log‐link function and compared with linear models.

**Results:**

The Spearman rank correlation coefficient between PFS/TTP and OS was 0.88 (95% confidence interval [CI] 0.85–0.89; *p* < 0.0001; *n* = 610) and between ORR and OS was 0.58 (0.52–0.64; *p* < 0.0001; *n* = 514). Model comparison favored the GLM model over the linear model, offering more accurate predictions for higher OS values. Consequently, PFS/TTP was the strongest predictor (pseudo‐*R* ^2^ = 0.75), with 1 added median PFS/TTP month associated with 13% (95% CI 13%–14%) increase in median OS. Subgroup analysis revealed that chemotherapy conferred significantly longer OS compared to targeted therapy in 1‐Agent and 2‐Agent trials, exhibiting a “very large” and “medium” effect size, respectively (rank biserial, *r*_rb_ = 0.40 [95% CI 0.22–0.56] and *r*_rb_ = 0.29 [0.16–0.41], both *p* < 0.0001), although inconsistent efficacy in 3‐Agent trials (*r*_rb_ = 0.12 [−0.07–0.30], *p* = 0.21).

**Conclusions:**

PFS/TTP is a more reliable surrogate than ORR and a strong predictor of OS in phase II trials of pancreatic cancer. Moreover, gamma GLM (log‐link function) is a robust tool for modeling positively skewed survival data with non‐constant variance, thus can be applied to other cancers' OS data of such nature.

## Graphical abstract

Progression‐free survival (PFS) is a strong predictor of overall survival (OS) in phase II trials of pancreatic ductal adenocarcinoma, with a Spearman rank correlation coefficient of 0.88. The study also found that chemotherapy is more effective than targeted therapy in improving OS in 1‐Agent and 2‐Agent trials, while the gamma generalized linear model (GLM) with a log‐link function is superior to linear models for predicting OS in positively skewed survival data.

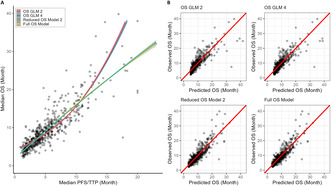

## 1 Introduction

Pancreatic ductal adenocarcinoma (PDAC) is a highly lethal gastrointestinal cancer that accounts for over 90% of pancreatic neoplasms. The one‐year overall survival (OS) rate for PDAC is 24%, with a dismal five‐year OS rate of just 9% [1, 2]. PDAC is often detected at late stages, limiting treatment options and leading to one of the lowest cancer survival rates. Current chemotherapeutic and targeted approaches have shown limited success compared to other cancer types.

Given the poor survival rates of PDAC, the evaluation of novel cancer drugs in clinical trials is crucial for improving the survival outcome. Clinical trials evaluating novel cancer drugs typically use OS as the primary endpoint to assess clinical effectiveness. However, surrogate endpoint of OS such as progression‐free survival (PFS) or time‐to‐progression (TTP) and overall response rate (ORR) are also being employed as they provide an early indication of the drug's efficacy [3]. These proxy endpoint are often attractive because they usually need smaller sample sizes, cost‐effective, quick to assess, and have lower susceptibility to confounding factors compared to OS. Therefore, surrogate endpoint are often preferred over clinical endpoint (OS) [4]. Two meta‐analyses [5, 6], comprising 30 and 50 randomized‐controlled trials (RCTs), testing only first‐line gemcitabine‐containing regimens in advanced pancreatic cancer reported correlation coefficients (Spearman rank) of 0.75 and 0.76 between PFS and OS, and preferred the use of PFS as a surrogate over ORR.

Nonetheless, many studies [7, 8, 9, 10, 11, 12, 13] have indicated that surrogate endpoint in oncology trials have little or no correlation to OS, and cancer drugs whose approval was expedited by surrogate endpoint failed to deliver promised efficacy in the real world. Makris et al. [14] in a meta‐analysis of 24 RCTs of comparable setting revealed a higher correlation between PFS and OS (Pearson, *r* = 0.86). This variability in correlation strength is likely due to differences in the method of correlation analysis and treatments in the experimental arm. Thus, additional studies are needed to validate the surrogacy of PFS/TTP and ORR. These studies will determine if the earlier findings translate into new trials of novel targeted agents and standard chemotherapy combinations. This validation is crucial, particularly in phase II trials due to their ample availability. Successful validation in phase II trials will facilitate the identification of effective treatment combinations in larger phase III trials and subsequent clinical practice.

The objective of clinical trials is to ensure safety and compare the efficacy of new treatment(s). Reliable predictions of efficacy and safety can help in designing trials and managing PDAC. For example, the incidence and severity of toxic effect were predicted by analyzing clinical trial data, where dose–volume of chemoradiation, age, sex, weight loss, and choice of chemotherapy were found significant in predicting the toxicity in PDAC [15, 16]. However, efficacy prediction in PDAC trials using OS data through appropriate statistical method has not been formally investigated, despite having a highly correlated surrogate PFS.

This paper explored 1867 efficacy data from phase II trials of PDAC to validate the surrogacy of PFS/TTP and ORR. Additionally, we developed different predictive OS models using PFS/TTP, treatment size, therapy type, and prior treatment history, and their performance was assessed through model comparison to identify optimal set of predictors.

## 2 Methods

### 2.1 Study Design

We collected 1867 efficacy data for phase II clinical trials of pancreatic adenocarcinoma from a total of 619 eligible articles with 776 treatment arms reporting efficacy. Efficacy data from each treatment arm in a trial were considered as a separate observation. The research design and workflow have been depicted in Figure S1.

### 2.2 Clinical Endpoint Extracted

The endpoint considered in this study were OS, PFS, TTP, and ORR. The median values of OS, PFS, and TTP were extracted in month, or converted to month if reported in days/weeks. The TTP was extracted when PFS was not reported and deemed as PFS in the study. The ORR consisted of partial response and/or complete response. Median disease‐free survival (DFS) was extracted for sensitivity analysis to assess model performance.

### 2.3 Predictor Variable Extracted

The predictor variables considered were therapy type, treatment size, PDAC stage, and previous treatment. The therapy type was divided into two types: chemotherapy and targeted therapy. Treatment size refers to the number of agents tested in a treatment arm where surgery and radiation therapy were counted. PDAC stage was roughly classified into early‐stage and advanced‐stage. Moreover, if PDAC stage and treatment history could not be determined due to ambiguity or absence of information, then considered as “undetermined,” and excluded from subgroup analyses. The detailed predictor selection criteria have been given in Table S1.

### 2.4 Search Strategy and Study Selection

On August 2022, relevant articles in the PubMed website were searched using a combination of MeSH terms and keywords consisting of “Pancreatic cancer” and “phase II clinical trial,” which returned a total of 1150 records after applying filter from year the 1992–2022 (detailed search strategy can be found in Table S2). Study eligibility criteria include phase II single‐arm or RCTs of early‐stage (resectable or borderline), non‐metastatic locally advanced, and advanced/metastatic pancreatic cancer testing cancer drug(s) efficacy. Trials reporting at least two of three clinical endpoint of interest (ORR, PFS/TTP, and OS) were included. In contrast, trials involving pancreatic neuroendocrine tumors (PNET), supportive therapies without anticancer agents, and vaccine therapy as a preventative measure were excluded from the study. Adjuvant/neoadjuvant trials reporting DFS were included for sensitivity analysis, however, excluded from the main analysis. From 619 included studies with 776 treatment arms, efficacy endpoint of interest and relevant predictor variables were collected. In December 2022, a total of 1867 efficacy data covering 33,238 patients were compiled in a worksheet (Table S3).

### 2.5 Data Extraction and Quality Assessment

Four of us (N.M.C., T.T.S.K., B.H.S., and N.A.T.) were involved in the process of data extraction and screening of the 1150 identified articles. The extracted information includes PDAC stage, previous treatment, number of participants, ORR, PFS, OS, combination size, therapy type, name of the drugs, and notes (Table S3). An in‐house protocol was developed for the data extraction process to facilitate uniform data collection among the four authors. Any discrepancy in the data extraction was discussed with FA and resolved by consensus. The overall process of data extraction was supervised by FA and ERK. Subsequently, data preprocessing following compilation was handled by FA. The curated dataset was then independently examined by HY and ERK for consistency.

### 2.6 Statistical Analysis

The correlation test between efficacy variables was Spearman rank correlation. The strength of association between OS, ORR, and PFS/TTP was assessed by Spearman's correlation coefficient (*r*_s_). The magnitude of correlation was deemed low, medium, and high if the *r*_s_ value lied between < 0.7, 0.7–0.85, and > 0.85, respectively. Consequently, a low, medium, and high correlation indicate lack of proof of validity of surrogate, unclear validity of surrogate, and proof of validity of surrogate, respectively [17]. The 95% CI for Spearman's correlation was calculated by Fieller et al. correction [18]. The OS was continuous and positively skewed non‐normally distributed variable which followed gamma distribution. Therefore, a gamma generalized linear model (GLM) with log‐link function was fitted to model the OS data. The least squares method was used to fit the OS data in multiple linear regression. The predictors were selected by the forward stepwise method and deemed significant at *p* value of 5%. The model with the smallest Akaike information criterion corrected (AICc) and Bayesian information criterion (BIC) was deemed superior in model comparison and prediction quality. The dispersion parameter (*φ*) of the gamma GLMs was calculated using Pearson chi‐squared statistic to assess the fit. To evaluate the generalizability of the candidate models to an unseen dataset, the whole dataset was divided by 80/20 train/test split and a 10‐fold cross‐validation was applied to train the model to minimize the bias of overfitting. Missing data were not imputed by any method. Additionally, sensitivity analysis was performed to check the robustness of the models.

The pooled median and 95% confidence interval (CI) of ORR, PFS, and OS were constructed by Hodges–Lehmann estimator. The test to compare the difference between group(s) was two‐sided Mann–Whitney or Kruskal–Wallis test at *p* < 0.05 significance. Post hoc Dunn's test was conducted for pair‐wise median comparison with Bonferroni–Holm adjustment. The effect size of group differences, rank biserial correlation coefficient (*r*_rb_) [19], and epsilon‐squared (ε^2^) [20] were interpreted by “effectsize” package [21]. All the analyses were carried out through R Statistical language v4.2.2 or higher using the packages: “sjPlot,” v2.8.15; “ggplot2,” v3.4.4; “ggstatsplot,” v0.12.1; “performance,” v0.10.5; “tidymodels” v1.1.1; and “effectsize,” v0.8.6.

## 3 Results

### 3.1 Pooled Median ORR, PFS/TTP, and OS

Our dataset of phase II PDAC trials contains 776 treatment arms from 619 studies, of which 180 and 593 arms are early‐stage and advanced, respectively. The pooled median ORR (*n* = 527), median PFS/TTP (*n* = 623), and median OS (*n* = 717) are 19% (95% CI 17.7–20.25), 4.85 months (4.6–5.1), and 8.47 months (8.15–8.8), respectively. The pooled median DFS (*n* = 46) and OS (*n* = 46) of neoadjuvant/adjuvant trials are 12.9 months (11.7–14.2) and 19.9 months (17.2–22.9), respectively.

### 3.2 PFS/TTP Is a Reliable Surrogate Than the ORR

Correlation analysis reveals that both the ORR and PFS/TTP positively correlate with OS. The PFS/TTP demonstrates a significant high positive correlation with OS, while ORR shows a significant low positive correlation (Spearman, *r*_s_ = 0.88 [95% CI 0.85–0.89; *n*_pairs_ = 610] and 0.58 [0.52–0.64; *n*_pairs_ = 514], respectively; both *p* < 0.0001). Consequently, these high and low correlations support the validity of PFS/TTP as a reliable surrogate while casting unclear validity of ORR in PDAC phase II trials.

### 3.3 PFS/TTP Is the Strongest Predictor of OS

#### 3.3.1 Gamma GLM

In our multivariate exploratory analysis, we performed model comparisons to prevent overfitting or underfitting of the OS data and identify optimal set of predictors. The outcome variable OS is a continuous and positively skewed data that follows a gamma distribution. Hence, we fitted a GLM with gamma family log‐link function to linearize the residuals. In line with the correlation finding, median PFS/TTP predicts the median OS strongly with a pseudo‐*R* ^2^ of 75.2% in OS GLM1 (Table 1). Moreover, the treatment size of the trial, the presence of targeted therapy in combination, and patient's previous treatment history contribute significantly to the model with minor improvement in pseudo‐*R* ^2^ in OS GLM2–4 (Table 1), indicating a better fit of the data. Nonetheless, these predictors considerably improve models' prediction quality by reducing AICc and BIC. However, the BIC does not favor OS GLM3 over OS GLM2. Notably, PDAC stage in OS GLM5 does not contribute significantly to the model (*p* = 0.125). Therefore, OS GLM4 is the candidate model with an optimal set of predictors. The coefficients of the predictors have been reported in model comparison in Table 1.

**TABLE 1:** Generalized linear OS model comparison. Gamma regression with a log‐link function was fitted to model the OS data. The order of the predictor variables in model comparison was selected by forward stepwise method with lowest AICc and BIC.

	OS GLM1		OS GLM2		OS GLM3		OS GLM4		OS GLM5	
Predictors	Estimates	95% CI	Estimates	95% CI	Estimates	95% CI	Estimates	95% CI	Estimates	95% CI
(Intercept)	4.42***	4.24–4.60	3.99***	3.79–4.20	4.09***	3.87–4.32	4.24***	4.00–4.50	4.27***	4.02–4.53
PFS/TTP^a^	1.13***	1.13–1.14	1.12***	1.11–1.13	1.12***	1.11–1.13	1.11***	1.11–1.12	1.11***	1.10–1.12
Treatment size			1.07***	1.05–1.09	1.07***	1.05–1.09	1.07***	1.05–1.09	1.07***	1.05–1.09
Therapy type [Targeted]					0.95*	0.91–0.99	0.95*	0.91–0.99	0.95*	0.91–1.00
Pretreated [Yes]							0.91***	0.87–0.96	0.91***	0.87–0.96
Stage [Early]									1.05 (ns)	0.99–1.11
Observations	610		610		610		599		597	
AICc	2711		2673		2669		2615		2608	
BIC	2724		2691		2691		2641		2639	
RMSE	4.19		3.60		3.54		3.50		3.44	
*R* ^2^ Nagelkerke	0.752		0.769		0.771		0.775		0.776	

Abbreviations: AIC^c^, Akaike information criterion corrected; BIC, Bayesian information criterion; CI, confidence interval; ns, not significant; OS, overall survival; PFS, progression‐free survival; RMSE, root mean squared error; TTP, time‐to‐progression.

^a^ Median TTP was regarded as median PFS in trials where median PFS was not reported.

* *p* < 0.05.

*** *p* < 0.001.

From Table 1, the OS GLM4 can be interpreted in the following manner. As the median PFS/TTP increases by 1 month, we expect the median OS to rise by 1.11 times (95% CI 1.11–1.12). Likewise, with an increase in treatment size, median OS increases by 1.07 fold (1.05–1.09). However, the use of targeted therapy instead of chemotherapy, as well as pretreatment of patients, both decrease the median OS by 5% (1%–9%) and 9% (4%–13%), respectively. The conditional prediction equation of “OS GLM4” for a trial testing targeted agent(s) in previously treated PDAC patients is as follows: “*Y* = 2.7183^(1.44551 + 0.10881 × *X*1 + 0.068495 × *X*2 − 0.05011 × *X*3 − 0.08999 × *X*4)^,” where *Y* = median OS (month), *X*_1_ = Median PFS/TTP (month), *X*_2_ = treatment size, *X*_3_ = therapy type [targeted], and *X*_4_ = pretreated [yes].

#### 3.3.2 Linear Regression Model

To assess how well gamma GLMs predict OS compared to ordinary regression, we constructed multiple linear regression model (Full OS Model). The full OS model contains all the predictors from the previous GLMs, and reduced OS models 1–3 have been sequentially developed by removing the statistically non‐significant predictors. The linear OS models have been compared in Table 2.

**TABLE 2:** Linear OS model comparison. The ordinary least squares method was used to fit the data.

	Full OS model		Reduced OS model 1		Reduced OS model 2		Reduced OS model 3	
Predictors	Estimates	95% CI	Estimates	95% CI	Estimates	95% CI	Estimates	95% CI
(Intercept)	1.52***	0.94–2.10	1.35***	0.85–1.85	1.26***	0.77–1.75	2.18***	1.79–2.56
PFS/TTP^a^	1.23***	1.15–1.31	1.23***	1.15–1.31	1.27***	1.20–1.34	1.37***	1.31–1.43
Treatment size	0.58***	0.37–0.79	0.59***	0.38–0.79	0.59***	0.39–0.80		
Therapy type [Targeted]	−0.30 (ns)	−0.75–0.14						
Pretreated [Yes]	−0.05 (ns)	−0.53–0.43						
Stage [Early]	0.61*	0.03–1.20	0.65*	0.08–1.23				
Observations	597		608		610		610	
*R* ^2^/*R* ^2^ adjusted	0.777/0.775		0.777/0.776		0.775/0.774		0.763/0.763	
RMSE	2.54		2.53		2.53		2.60	
AICc	2821		2862		2873		2902	
BIC	2851		2884		2890		2915	

Abbreviations: AICc, Akaike information criterion corrected; BIC, Bayesian information criterion; CI, confidence interval; ns, not significant; OS, overall survival; PFS, progression‐free survival; RMSE, root mean squared error; TTP, time‐to‐progression.

^a^ Median TTP was regarded as median PFS in trials where median PFS was not reported.

* *p* < 0.05.

*** *p* < 0.001.

Although the goodness of the fit, AICc and BIC, favor the full OS model, the coefficient of determination (adjusted R^2^) supports the Reduced OS Model 2 over the full OS model (Table 2). Moreover, the adjusted *R* ^2^ of the two models is marginally different (77.5% vs 77.4%).

Therefore, the preference leans toward the simpler of the two models. PDAC stage has been skipped from the Reduced OS Model 2 due to its minimal impact on the adjusted *R* ^2^ and wide CI, indicating higher uncertainty. The linear predictor of the “Reduced OS Model 2” is as follows: “*Y* = 1.26 + 1.27 × *X*_1_ + 0.59 × *X*_2_,” where *Y* = median OS (month), *X*_1_ = median PFS/TTP (month), and *X*_2_ = treatment size.

**FIGURE 1:**
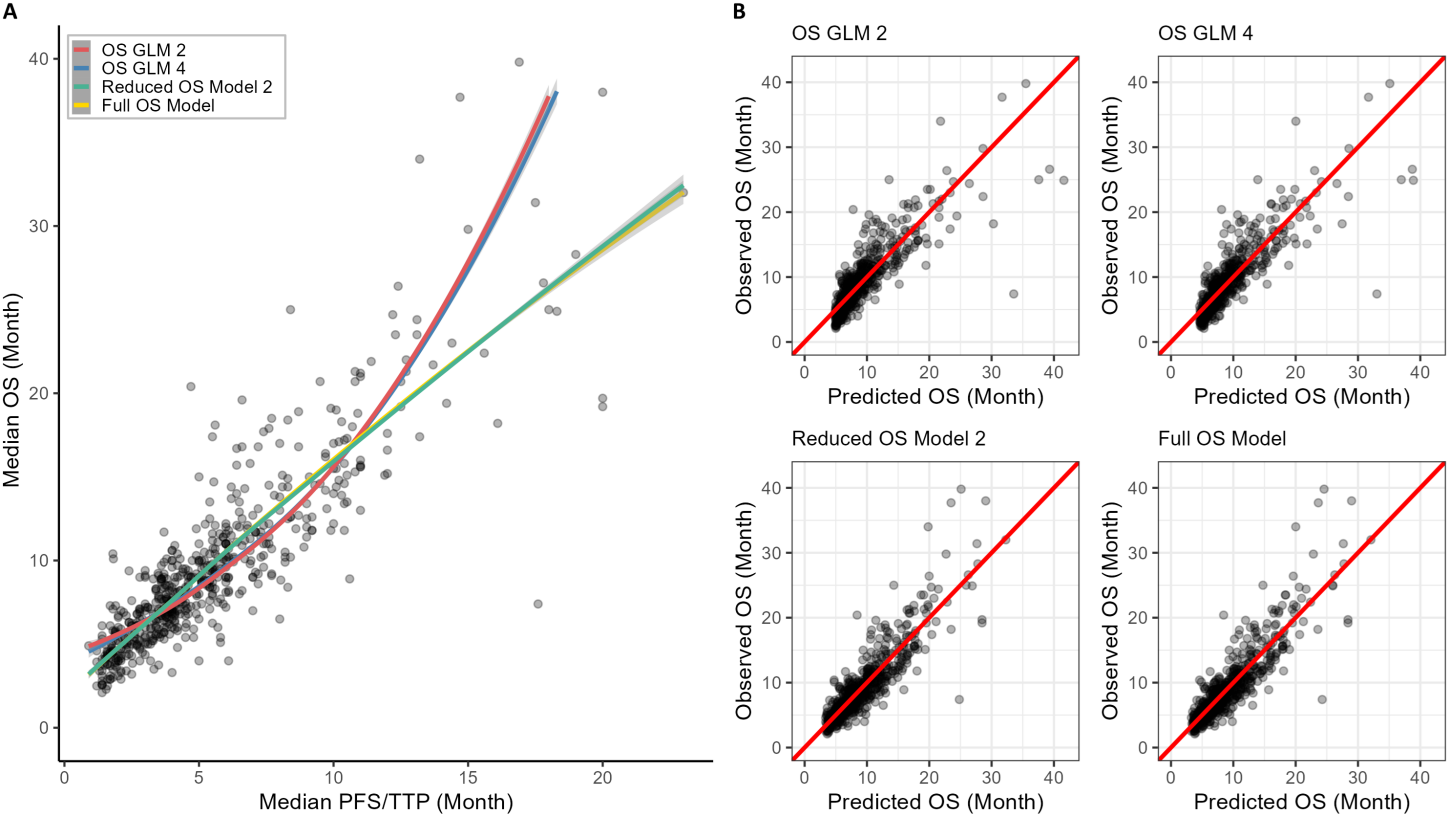
Candidate overall survival (OS) models. (A) Scatterplot of positive relationship between median OS and median progression‐free survival (PFS) or time‐to‐progression (TTP), along with the fitted curves and lines from significant candidate gamma GLMs with a log‐link function (OS GLM2 and OS GLM4) and ordinary linear regression (Reduced OS Model 2 and Full OS Model). (B) Plots of observed versus model‐predicted OS data of corresponding candidate models. The diagonal line signifies perfect agreement between predicted and observed OS. OS GLM2 consists of “median PFS/TTP” and “treatment size” as the predictors (*n* = 610); OS GLM4 consists of OS GLM2 predictors plus “therapy type” and “previous treatment” (*n* = 599); Reduced OS Model 2 consists of OS GLM2 predictors (*n* = 610); Full OS Model consists of OS GLM4 predictors plus “PDAC stage” (*n* = 597).

Figure 1 illustrates a scatterplot of strong positive relation between OS and PFS, along with the fitted curves and lines from significant candidate gamma GLMs (OS GLM2 and GLM4) and ordinary regression (Full OS Model and Reduced OS Model 2), and their corresponding plots of observed versus model‐predicted OS data.

Although all candidate models predict OS with higher accuracy, the predicted OS values become gradually inconsistent when the OS values exceed 20 months (Figure 1B). This high predictive accuracy and deviation at higher values are also reflected in the “posterior predictive check” and “homogeneity of variance” plots, as shown in residual plots (Figures S2 and S3). These residual plots reveal a considerable amount of non‐constant variance at higher predictor levels, while residuals appear to follow an approximately normal distribution and low collinearity between predictors.

Overall, both the OS GLMs and linear OS models exhibit pairwise similarity in terms of prediction accuracy; however, the OS GLM4 demonstrates slightly better performance in handling outliers. Therefore, linear model performs better at describing or predicting our current dataset, while the OS GLM4 offers greater robustness and is preferred for generalizing or predicting future OS, especially in cases with higher variability. Additionally, the linear OS model is favored when the median PFS/TTP of a trial is less than 12 months.

### 3.4 Generalizability of the Model

Subsequently, we compared the generalizability of “OS GLM2” and “Reduced OS Model 2” on a new dataset, as they contain the same predictors. We divided the dataset into 80/20 train/test split and trained the models using 10‐fold cross‐validation. Interestingly, we found that “OS GLM2” performs better in predicting OS, with an *R* ^2^ of 80.8% and a Root Mean Squared Error (RMSE) of 2.10, compared to “Reduced OS Model 2,” which has an *R* ^2^ of 78.4% and an RMSE of 2.16. This indicates that the gamma GLMs provide a better goodness of fit and higher prediction accuracy for skewed survival dataset. The model evaluation metrics for the train and test dataset can be found in Table S4. This generalizability highlights gamma GLMs robustness in predicting non‐negative continuous and right‐skewed OS data and potential application in other cancer types.

### 3.5 Impact of Treatment Size and Therapy Type

**FIGURE 2:**
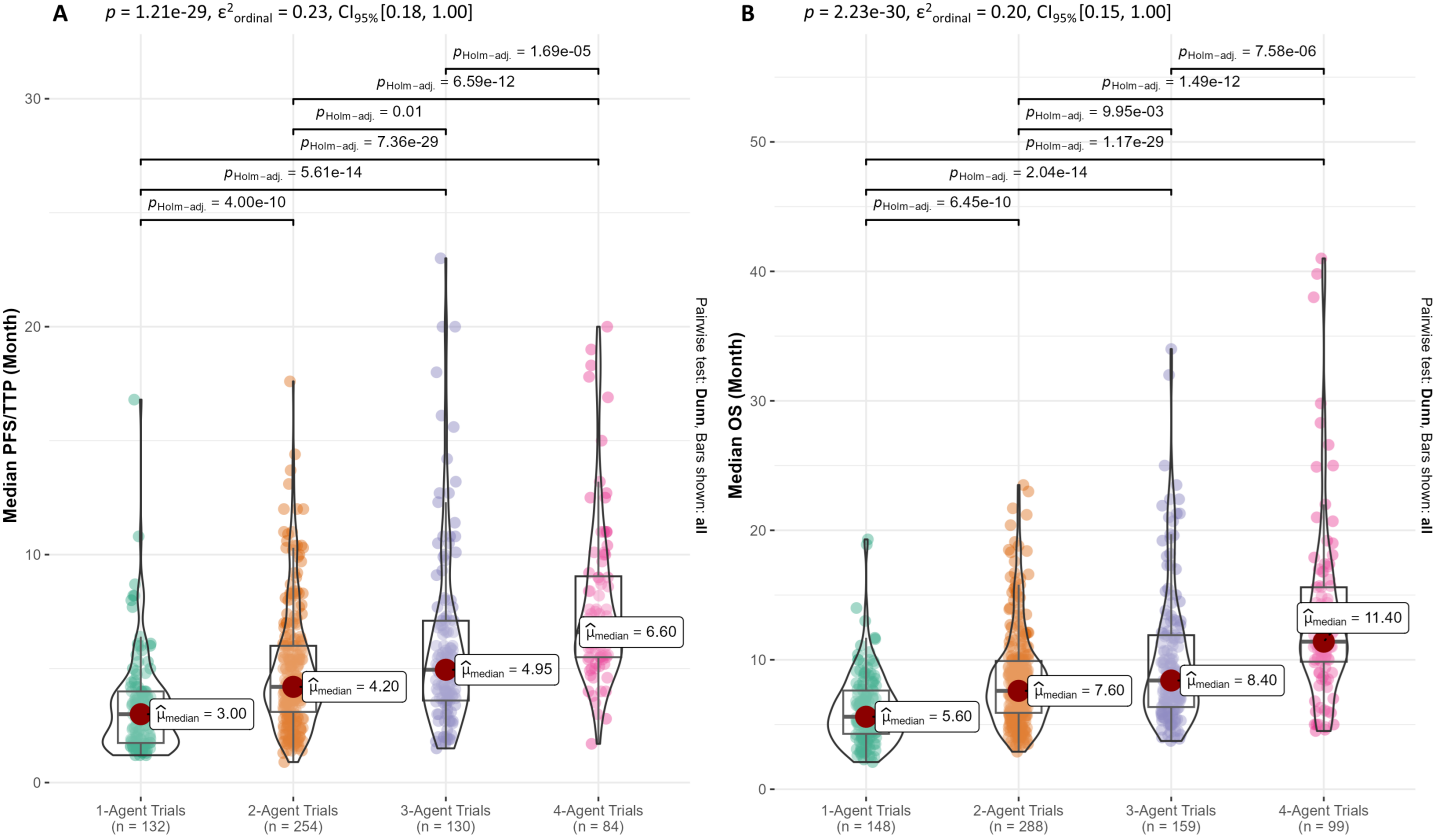
Impact of treatment size on efficacy endpoint of phase II PDAC trials. (A) Box/violin plots of median progression‐free survival (PFS) or time‐to‐progression (TTP) of trials binned according to treatment size. (B) Box/violin plots of median overall survival (OS) of trials binned according to treatment size. Differences between group medians were compared by Kruskal–Wallis test at *p* < 0.05 significance. Post hoc Dunn's test with Bonferroni–Holm correction was performed for pairwise median comparisons. The effect size of the difference was assessed by epsilon‐squared (ε^2^).

**FIGURE 3:**
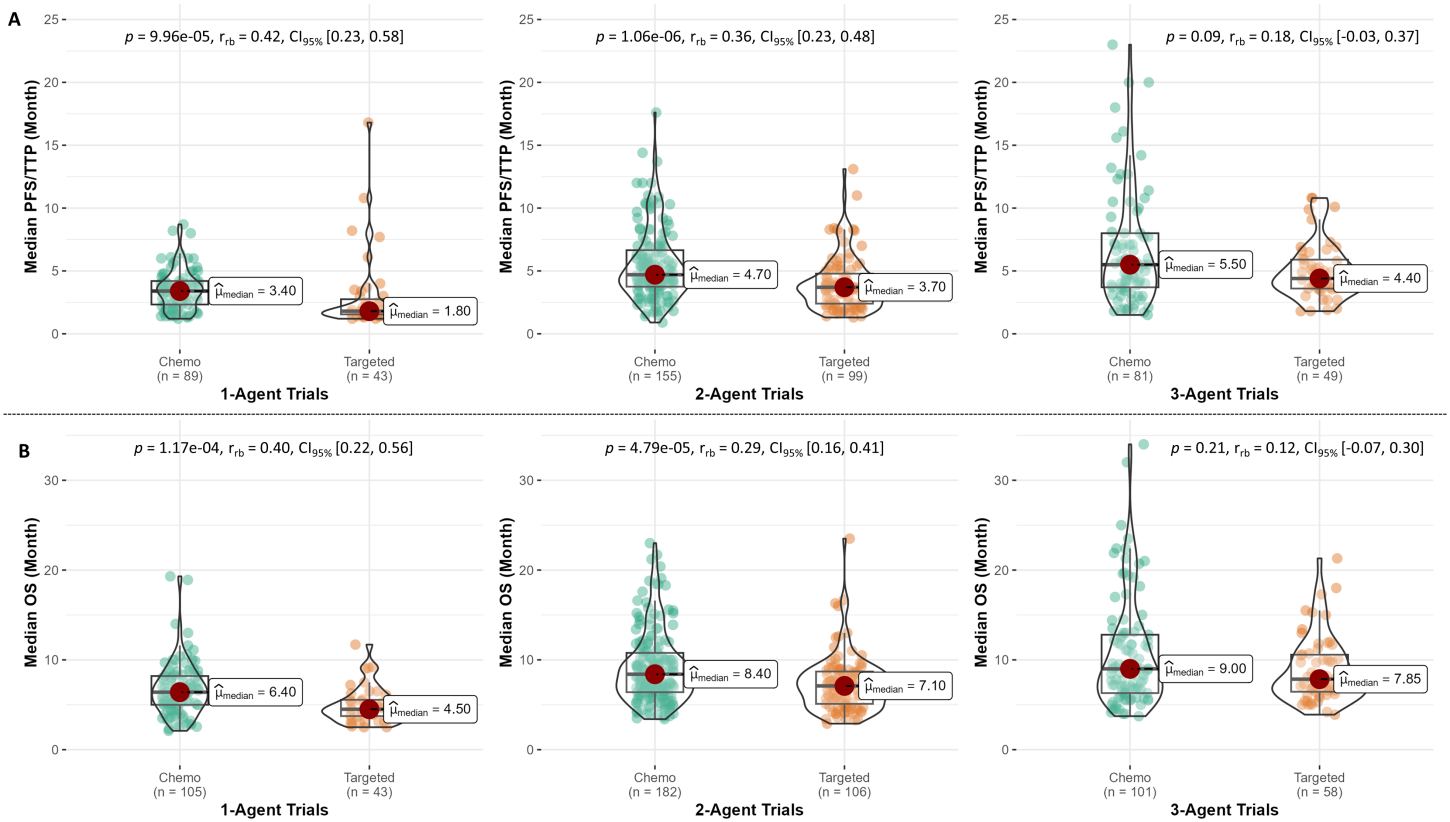
Impact of chemotherapy versus targeted therapy on efficacy endpoint of PDAC phase II trials. (A) Box/violin plots of median progression‐free survival (PFS) or time‐to‐progression (TTP) of chemotherapy (chemo) and targeted therapy trials of comparable treatment size. (B) Box/violin plots of median overall survival (OS) of chemotherapy and targeted therapy trials of comparable treatment size. Targeted therapy trials contain at least one targeted agent(s) in combination with or without chemotherapy. A targeted agent includes kinase inhibitors or cancer immunotherapies. Difference between group ranks was compared by two‐sided Mann–Whitney test at 5% significance. The effect size of the rank difference was assessed by rank biserial correlation coefficient (*r*_rb_). 4‐Agent trials were skipped due to small sample size (*n* < 30).

We observed that treatment size had a significant positive effect on the OS across all candidate models, while the impact of therapy type remains unclear, possibly due to its variable interactions among different treatment sizes. Consequently, we investigated the impact of number of agents in combination on PFS/TTP and OS (Figure 2) and determined the effect size of therapy type on PFS/TTP and OS at different treatment sizes (Figure 3). As the number of agents increases, both the median PFS/TTP and OS exhibit a corresponding upward trend. Furthermore, the medians between groups differ significantly with a large effect size for both PFS/TTP and OS (epsilon‐squared, ε^2^ = 0.23 [95% CI 0.18–1.00] and ε^2^ = 0.20 [0.15–1.00], respectively, both *p* < 0.0001). Subsequently, post hoc pairwise Dunn's tests confirm statistical differences among all group medians, as shown in Figure 2.

Figure 3 illustrates the impact of chemotherapy and targeted therapy on comparable treatment size. For instance, in 1‐Agent trials, chemotherapy exhibits a significant increase in PFS/TTP and OS, compared to targeted therapy, with a “very large” effect size (rank biserial, *r*_rb_ = 0.42 [95% CI 0.23–0.58] and *r*_rb_ = 0.40 [0.22–0.56], respectively, both *p* < 0.0001). Additionally, 2‐Agent chemotherapy trials show a significant positive “large” effect size for PFS and a “medium” effect size for OS compared to 2‐Agent targeted therapy trials (r_rb_ = 0.36 [0.23–0.48] and *r*_rb_ = 0.29 [0.16–0.41], respectively, both *p* < 0.0001). Furthermore, these findings also resonate with the inferences derived from the “OS GLM4” model that targeted therapy slightly negates the OS if replaced by chemotherapy. However, the efficacy of chemotherapy and targeted therapy in 3‐Agent trials remains inconclusive as the rank differences are not statistically significant, and the lower bound of the 95% CI for effect size slightly falls into the negative range (PFS: *r*_rb_ = 0.18 [−0.03–0.37], *p* = 0.09; OS: *r*_rb_ = 0.12 [−0.07–0.30], *p* = 0.21), as shown in Figure 3. Altogether, PFS/TTP and OS increase significantly for up to 4‐Agent trials and chemotherapy confers longer survival in 1‐Agent and 2‐Agent PDAC phase II trials.

## 4 Discussion

Our analysis demonstrates a high positive correlation between PFS/TTP to OS (*r*_s_ = 0.88, *p* < 0.0001) and a low correlation between ORR to OS (*r*_s_ = 0.58, *p* < 0.0001). This finding favors PFS/TTP as a reliable surrogate endpoint in phase II trials of PDAC. A positive correlation between PFS and OS was expected; however, the strength of the association between endpoint is difficult to estimate. For example, a meta‐analysis [14] of 24 phase III RCTs of pancreatic cancer reported PFS and OS correlation of 0.86 (*p* < 0.0001), which is similar to our finding. However, the association strength between ORR and OS was lower in that meta‐analysis (*r* = 0.45, *p* = 0.02), which further reinforces the use of PFS over ORR. Moreover, two additional studies combining 80 RCTs of pancreatic cancer reported slightly lower correlation (0.75 and 0.76) between PFS and OS [5, 6]. These prior studies focused on first‐line RCTs with gemcitabine in the control arm, whereas our pooled analysis encompassed all available phase II trials to date, incorporating a diverse range of treatments in both randomized and non‐randomized single‐arm trials. Furthermore, in non‐pancreatic solid tumors, the validity of surrogacy of PFS resonates with our finding, with correlation coefficients of 0.82 and 0.84 [22, 23].

Both the OS models (GLM and linear) predict the OS values with a maximum of 77% accuracy, which is relatively high. This accuracy is due to the high correlation between PFS/TTP and OS and large number of sample size (*n*_pairs_ = 610). Moreover, the residual plots of the two models in Figures S2 and S3 are similar with nearly normally distributed residuals, no dependency or pattern in the residuals, and low collinearity with variance inflation factor (< 2) between predictors for all models. However, homogeneity of variance plots of the models in Figures S2 and S3 indicates considerable heteroscedasticity of the residuals, meaning that variances of residuals are not constant, and spread of the residuals becomes inconsistent with higher PFS/TTP values. This is evident in the findings where model‐predicted OS values deviate from high observed OS values, particularly when the observed OS values are high (OS > 20 months).

The mean–variance relationship of the response variable is one of the key assumptions of the gamma GLM. The dispersion parameter (*φ*) models the relationship between mean (μ) and variance in a GLM. The dispersion ratio for both candidate models “OS GLM2” and “OS GLM4” was 1.4, indicating slight overdispersion. These statistically significant results (both *p* < 0.05) suggest that the observed variance exceeds the expected variance, indicating extra variability not accounted for by the model's current structure. To address this, we compared the performance of our gamma “OS GLM2” [var(Y) ∞ *φ*μ^2^] model with quasi‐GLMs (log‐link) employing different variance structure [var(Y) ∞ *φ*μ] and [var(Y) ∞ *φ*μ^3^]. We maintained an 80/20 train‐test split and utilized 10‐fold cross‐validation to train the quasi‐models with the same set of predictors used in the gamma “OS GLM2.” Interestingly, the performance metrics, specifically *R* ^2^ and RMSE, were marginally better for the gamma GLM compared to the quasi‐GLMs (results not shown). These results imply that the gamma GLM is robust for modeling positively skewed OS data and performs well even when the mean–variance assumptions are not perfectly met. Since the overdispersion is slight and the dispersion ratio is below 1.5, we determined that our inferential statistics remain robust, and the impact of overdispersion on model performance for a new dataset is minimal. Nonetheless, we acknowledge this as a limitation of our model and suggest that future studies consider models that can better accommodate potential overdispersion.

Sensitivity analysis revealed that the exclusion of TTP endpoint from the model did not change the model performance noticeably and significant predictors remained same in both model types. For example, the estimates of predictors in the GLMs remained the same as previously, with a pseudo‐*R* ^2^ of 76%–78%. This is because the correlation strength between TTP and OS is similar to that between PFS and OS (*r*_s_ = 0.87, *p* < 0.0001, *n*_pairs_ = 107), which validates the interchangeability of TTP. In contrast, inclusion of 46 DFS endpoint to the PFS/TTP had no impact on the correlation to OS (*r*_s_ = 0.88, *p* < 0.0001), despite DFS having a median value of almost 2.6 times larger than PFS/TTP (12.9 vs 4.85). However, DFS alone displayed a significantly low correlation with OS (*r*_s_ = 0.75, *p* < 0.0001). Moreover, the addition of DFS into PFS/TTP slightly increased model performance in OS GLM model comparison with a pseudo‐*R* ^2^ ranging from 78% to 79%, however, the number of significant predictors remained same (data not shown). In contrast, the performance of linear model did not drop substantially by adding DFS, for example, adjusted *R* ^2^ remained at 76%. This implies that OS GLM models can fit the high‐value OS data better than linear OS models.

The predictors analyzed in our study could not explain all the variances associated with the survival of patients. Other predictors such as patient's age, ECOG performance status, and metastatic site were found to be significant predictors of survival in pancreatic cancer [24, 25, 26]. Inclusion of these predictors could reduce the errors in explaining the OS and improve model performance. Moreover, all trials were considered equal in weight in our analysis. Furthermore, the ORR was omitted as a model predictor due to the low number of pairwise observations with OS and PFS/TTP. We also did not analyze interaction effects among predictors in our model which could improve the predictability, for example, there could be a significant interaction between PFS/TTP and ORR. Moreover, our findings might be influenced by publication bias, given that negative outcomes are less frequently published. Nonetheless, we consider our work to be the first in this clinical setting of phase II trials. Therefore, generalizing the model to large sample phase III trials of pancreatic cancer might produce unsatisfactory results, necessitating careful interpretation.

We observed chemotherapy conferring prolonged survival than targeted therapy in 1‐Agent and‐2‐Agent trials (Figure 3; OS GLM3–4, Table 1). Conversely, it was not clear as to whether replacing chemotherapy by a targeted agent in 3‐Agent trials extends the PFS/TTP and OS. This difference in efficacy is possibly because there could be interactions between agents that potentially negate the combined efficacy in 3‐Agent trials or diminish the effectiveness of the substituted targeted agent. Alternatively, the sample size of 3‐Agent trials may have failed to detect a small difference. In concordance with our findings, many RCTs testing efficacy of different targeted agents in pancreatic cancer reported that the addition of targeted therapy to chemotherapy does not significantly improve the OS of patients [27, 28, 29, 30, 31, 32, 33], as opposed to one double‐blind phase III RCT conducted by Moore et al. [34], demonstrated statistically significant improved survival conferred by targeted agent.

Our pooled analysis highlighted an increasing trend in survival with increasing number of agents in the trial (Figure 2). Moreover, our study was focused on the efficacy side of the therapy, overlooking the toxicity or quality aspect of the life. For instance, a standard 4‐agent combination (FOLFIRINOX) substantially extended PDAC survival in a feasible subgroup; however, at the expense of higher level of toxicity compared to other therapies [35, 36]. Additionally, a larger treatment size does not necessarily extend PFS and OS, as reported in a meta‐analysis comparing modified FOLFIRINOX versus gemcitabine plus nab‐paclitaxel, where both treatments had similar survival and toxicity profile in advanced PDAC [37]. Furthermore, RCTs of PDAC confirmed that patients without chemoradiation therapy exhibited favorable OS in adjuvant setting. However, in nonrandomized trials containing similar patient subtypes, radiation therapy enhanced the effectiveness of chemotherapy [38, 39, 40, 41]. These findings suggest that adding more agents to a treatment regimen may not always lead to improved outcomes.

Our study design had a caveat in differentiating between early and advanced disease. We roughly classified the stage into two general types and relied primarily upon the title of the study specifying the stage or TNM staging from patient characteristics. However, it is difficult to segregate early‐stage tumors from advanced‐stage, especially in trials that recruited a blend of borderline resectable pancreatic cancer (BRPC) and locally advanced pancreatic cancer (LAPC), and TNM stage II and III patients. In our design, we grouped resectable, BRPC, and LAPC as early‐stage to address the challenge of isolating BRPC and LAPC. However, unresectable LAPC is sometimes categorized as advanced in certain settings [26]. Preferably, LAPC should fall between early and advanced stages in terms of disease severity. Nonetheless, a group of experts recommended that the LAPC should not be considered as advanced cancer and they must be examined separately in clinical trials [42]. Moreover, LAPC patients were separated from the advanced due to a notable difference in OS, with advanced/metastatic patients exhibiting significantly shorter median OS compared to LAPC [35, 36, 43, 44, 45, 46].

## 5 Conclusions

This is the first study pooling together a large number of phase II trials to assess the surrogacy of PFS/TTP and ORR in pancreatic cancer and to explore the predictive variables of OS. Additionally, our study represents an innovative approach to analyzing and predicting OS data through the integration of a suitable GLM, and model comparison to identify optimal set of predictors. Although response rate delivers a quick measure of efficacy, our analysis provides compelling evidence that PFS/TTP is a stronger surrogate for OS than ORR. Therefore, we recommend using PFS/TTP as the primary endpoint in phase II trials of pancreatic adenocarcinoma. Our study also sheds light on how the predictive factors influence OS in PDAC by utilizing a blend of generalized and ordinary linear models, where the PFS/TTP emerged as the most significant predictor of OS. Subgroup analysis revealed that combining more agents enhances survival, with chemotherapy demonstrating superior efficacy over targeted therapy in 1‐Agent and 2‐Agent trials. However, this efficacy is unclear for 3‐Agent trials, highlighting the need for careful assessment when combining targeted agents with standard chemotherapy. Nonetheless, the potential of targeted therapy in combination therapies for pancreatic cancer remains promising.

## Author Contributions

**Eva Rahman Kabir:** conceptualization (equal), funding acquisition (lead), investigation (equal), project administration (lead), resources (equal), supervision (lead), writing – original draft (equal), writing – review and editing (equal). **Faruque Azam:** conceptualization (equal), data curation (lead), formal analysis (lead), investigation (equal), methodology (lead), software (lead), validation (equal), visualization (lead), writing – original draft (lead), writing – review and editing (equal). **Tanisha Tabassum Sayka Khan:** formal analysis (equal), validation (equal), writing – original draft (equal), writing – review and editing (equal). **Hasina Yasmin:** supervision (equal), validation (equal), writing – review and editing (equal). **Namara Mariam Chowdhury:** formal analysis (equal), investigation (equal), methodology (equal), validation (equal). **Syeda Maliha Ahmed:** data curation (supporting), writing – original draft (equal). **Baejid Hossain Sagar:** data curation (supporting), writing – original draft (equal). **Nasrin Ahmed Tahrim:** data curation (supporting), writing – original draft (equal).

## Conflicts of Interest

The authors declare no conflicts of interest.

## Supporting information


Figure S1.

Figure S2.

Figure S3.

Table S1.

Table S2.

Table S3.

Table S4.

## Data Availability

The data that supports the findings of this study are available in the supporting information of this article.
