# Supplementary material for: Modeling Overall Survival in Patients With Pancreatic Cancer From a Pooled Analysis of Phase II Trials
Source: Cancer Med. 2024 Oct 10;13(19):e70289. doi: 10.1002/cam4.70289 (PMC11465028; doi:10.1002/cam4.70289)

**Figure S1.** A flow chart depicting the study design. PNET, Pancreatic Neuroendocrine Tumors; PDAC, Pancreatic Ductal Adenocarcinoma; ORR, overall response rate; PFS, progression-free survival; TTP, time-to-progression; OS, overall survival; GLM, generalized linear model.


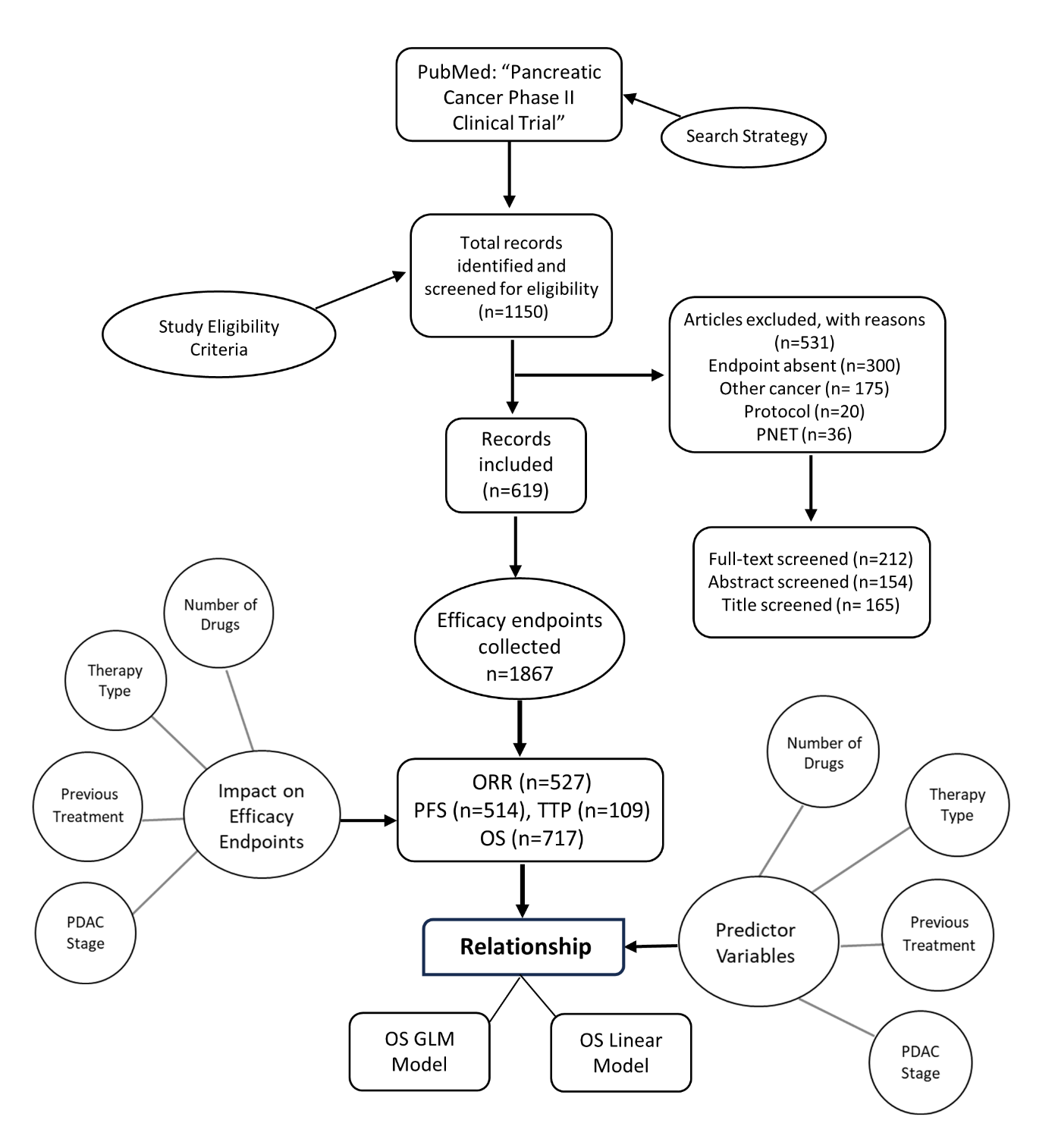

Supplement: Supplementary file 1 — Figure S1. [file CAM4-13-e70289-s002.docx]
