# Supplementary material for: Modeling Overall Survival in Patients With Pancreatic Cancer From a Pooled Analysis of Phase II Trials
Source: Cancer Med. 2024 Oct 10;13(19):e70289. doi: 10.1002/cam4.70289 (PMC11465028; doi:10.1002/cam4.70289)
Supplement: Supplementary file 2 — Figure S2. [file CAM4-13-e70289-s007.docx]

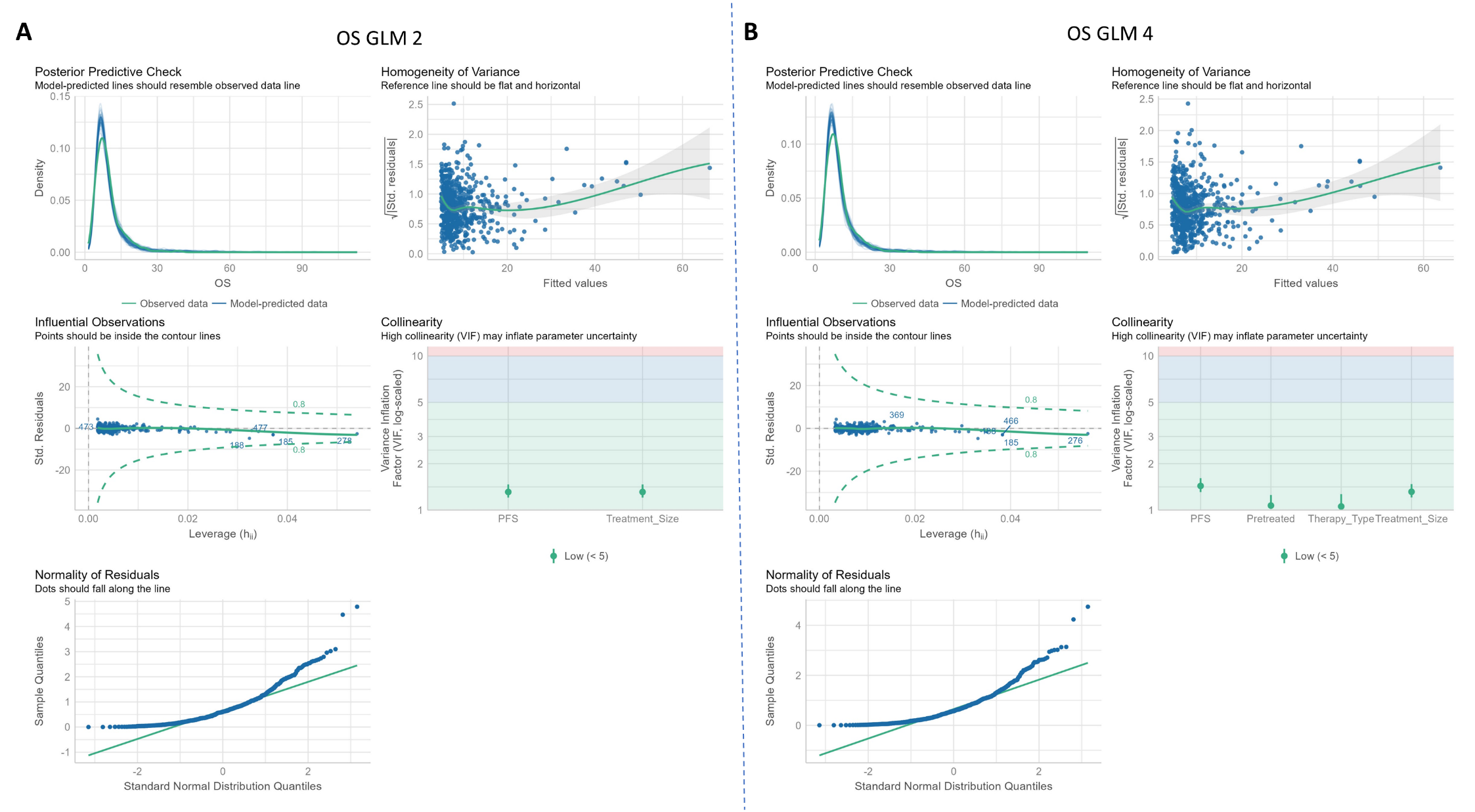
**Figure S2.** Residual diagnostic plots of gamma generalized linear OS models with a log-link function. **(A)** OS GLM2 model consisting of “median PFS/TTP” and “treatment size” as predictors of OS. **(B)** OS GLM4 model consisting of “median PFS/TTP”, “treatment size”, “therapy type”, and “previous treatment” as predictors.
