# Supplementary material for: Modeling Overall Survival in Patients With Pancreatic Cancer From a Pooled Analysis of Phase II Trials
Source: Cancer Med. 2024 Oct 10;13(19):e70289. doi: 10.1002/cam4.70289 (PMC11465028; doi:10.1002/cam4.70289)

**Figure S3.** Residual diagnostic plots of ordinary linear OS model. **(A)** Reduced OS Model 2 consisting of “median PFS/TTP”,” treatment size” as predictors of OS. **(B)** Full OS Model consisting of “median PFS/TTP”, “treatment size”, “therapy type”, “previous treatment”, and “PDAC stage” as predictors.


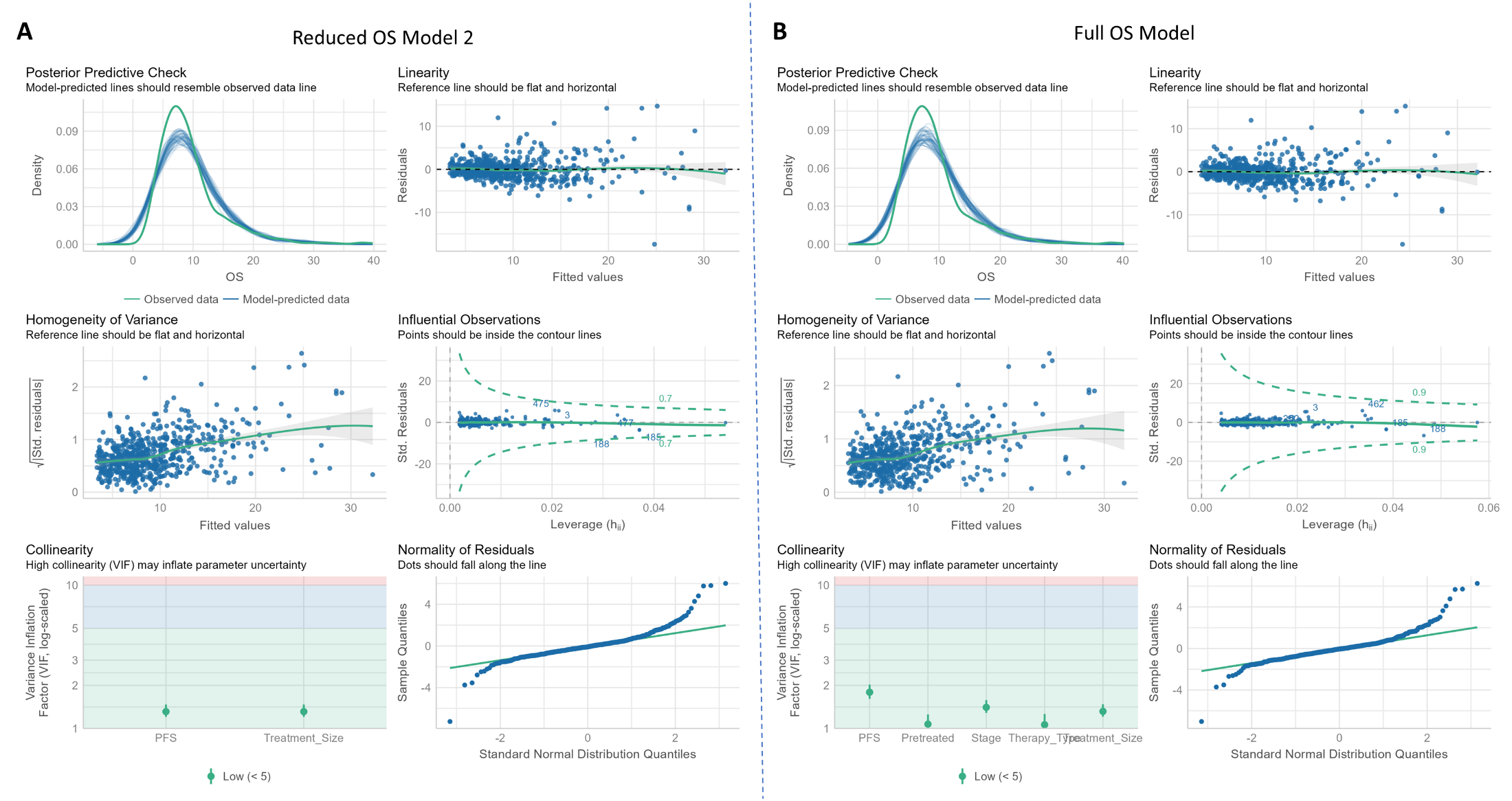

Supplement: Supplementary file 3 — Figure S3. [file CAM4-13-e70289-s005.docx]
