# Supplementary material for: Modeling Overall Survival in Patients With Pancreatic Cancer From a Pooled Analysis of Phase II Trials
Source: Cancer Med. 2024 Oct 10;13(19):e70289. doi: 10.1002/cam4.70289 (PMC11465028; doi:10.1002/cam4.70289)
Supplement: Supplementary file 4 — Table S1. [file CAM4-13-e70289-s003.docx]

| **Table S1.** Selection criteria of extracted predictor variables from phase II clinical trials of pancreatic adenocarcinoma. | |
| --- | --- |
| **Predictor Variable** | **Selection Criteria** |
| Therapy type | Therapy type was considered as a predictor variable of efficacy. Therapies in a trial were roughly classified either as “chemotherapy” or “targeted therapy”. All the chemotherapies such as alkylating agents, cytotoxic drugs, antimetabolites, cytotoxic alkaloids, antitumor antibiotics were considered as chemotherapy. Alternatively, kinase inhibitors, modern immunotherapies, and agents targeting against any specific protein were classified as targeted therapy. However, surgery and radiation therapy were regarded as non-targeted agents, thereby taken under the chemotherapy group. |
| Treatment size | Combination size or number of drugs/agents in a trial refers to how many agents were used in the trial as an intent to cure cancer and considered a significant predictor of efficacy. For example, steroids used to manage cancer associated pain, agents to counteract chemotherapy induced leukopenia, and supplements to reduce toxicity were not counted as an agent. However, leucovorin or folinic acid was counted in the combination, as it is widely used in standard chemotherapy combinations and enhances effectiveness of the therapy. Vaccines used to prevent cancer was not counted as an agent, otherwise, if targeted against any tumour protein/antigen was counted as a targeted agent. Moreover, if surgery and/or radiation therapy were performed in a trial, it was counted as an agent to the combination size. |
| PDAC stage | PDAC stage in a trial refers to the stage of the recruited patients’ pancreatic adenocarcinoma. This was roughly divided into two categories: “early-stage” and “advanced”. If most of the recruited patients’ tumour were metastatic, recurrent, or advanced, then the stage was deemed as advanced stage. In contrast, TNM stage I/II, resectable, borderline resectable, and locally advanced PDAC were counted as early-stage. However, TNM stage III/IV or IIIb with nodal involvement were deemed advanced. Moreover, If the stage could not be determined from patient characteristics section due to ambiguity or absence of information, then the stage was considered “undetermined” and excluded from subgroup analysis. |
| Previous treatment | Previous treatment history of the patients in a trial was considered another important predictor variable of efficacy. If the patients received therapy prior to the recruitment to the trial considered as “previously treated”, whereas if recruited as first-line therapy or chemotherapy naïve then deemed as “previously untreated”. However, if such information could not be retrieved from the study, then treatment history was deemed as “undetermined” and excluded from subgroup analysis. |
