# Supplementary material for: Modeling Overall Survival in Patients With Pancreatic Cancer From a Pooled Analysis of Phase II Trials
Source: Cancer Med. 2024 Oct 10;13(19):e70289. doi: 10.1002/cam4.70289 (PMC11465028; doi:10.1002/cam4.70289)
Supplement: Supplementary file 5 — Table S2. [file CAM4-13-e70289-s004.docx]

| **Table S2.** PubMed search strategy | | |
| --- | --- | --- |
| **Sl.** | **Concept** | **MeSH Terms and Keywords** |
| #1 | Pancreatic Cancer | "Pancreatic Neoplasms/drug therapy"[Mesh] OR “Pancreatic Cancer”[tiab] OR “Pancreatic Adenocarcinoma”[tiab] OR “Pancreatic Ductal Adenocarcinoma”[tiab] OR “Pancreatic Neoplasm*”[tiab] OR “Pancreatic Carcinoma”[tiab] OR “Pancreatic Malignancy”[tiab] |
| #2 | Phase II Clinical Trial | "Clinical Trial, Phase II" [Publication Type] OR “Phase II clinical trial” OR “phase II trial” OR “Phase 2 clinical trial” OR “phase 2 trial” |
| #3 |  | #1 AND #2 Filters: from 1992 - 2022. |
