# Supplementary material for: Modeling Overall Survival in Patients With Pancreatic Cancer From a Pooled Analysis of Phase II Trials
Source: Cancer Med. 2024 Oct 10;13(19):e70289. doi: 10.1002/cam4.70289 (PMC11465028; doi:10.1002/cam4.70289)
Supplement: Supplementary file 7 — Table S4. [file CAM4-13-e70289-s001.docx]

| **Table S4.** Model evaluation metrics. The dataset (n=610) was split by 80/20 ratio and a 10-fold cross-validation was applied to minimize the bias of overfitting. The median PFS/TTP and treatment size were predictors in both models. | | | | | | | | | |
| --- | --- | --- | --- | --- | --- | --- | --- | --- | --- |
|  | | ^†^**Reduced OS Model 2** | | | | | ^‡^**OS GLM2** | | |
|  | Metric | | Mean | | N | Standard Error | Mean | N | Standard Error |
| Training Dataset (n=486) | MAE | | 1.70 | | 10 | 0.0606 | 2.04 | 10 | 0.0855 |
|  | RMSE | | 2.62 | | 10 | 0.149 | 3.65 | 10 | 0.412 |
|  | R^2^ | | 0.784 | | 10 | 0.0218 | 0.729 | 10 | 0.0365 |
|  | Metric | | | Estimate | | | Estimate | | |
| Test Dataset (n=124) | MAE | | 1.62 | | | | 1.61 | | |
|  | RMSE | | 2.16 | | | | 2.10 | | |
|  | R^2^ | | 0.784 | | | | 0.808 | | |
| ^†^ Ordinary least squared regression model  ^‡^ Gamma generalized linear model with a log-link function  MAE, mean absolute error; RMSE, root mean squared error. | | | | | | | | | |
